# Disparities in the Evolution of the COVID-19 Pandemic between Spanish Provinces

**DOI:** 10.3390/ijerph18105085

**Published:** 2021-05-11

**Authors:** Héctor López-Mendoza, Antonio Montañés, F. Javier Moliner-Lahoz

**Affiliations:** 1 Directorate-General of Public Health, Aragon Department of Health, 50017 Zaragoza, Spain; hlopezmendoza@salud.aragon.es; 2 Preventive Medicine and Public Health Department, Lozano Blesa University Hospital, 50009 Zaragoza, Spain; jmoliner@unizar.es; 3 Economic Analysis Department, University of Zaragoza, 50005 Zaragoza, Spain

**Keywords:** epidemiology, convergence, incidence, SARS-CoV-2 infection, COVID-19, Spain

## Abstract

Spain experienced a second wave of the COVID-19 pandemic in autumn 2020, which has been approached with different measures by regional authorities. We analyze the presence of convergence in the cumulative incidence for 14 days (CI_14_) in provinces and self-governing cities. The Phillips–Sul methodology was used to study the grouping of behavior between provinces, and an ordered logit model was estimated to understand the forces that drive creating the different convergence clubs. We reject the presence of a single pattern of behavior in the evolution of the CI_14_ across territories. Four statistically different convergence clubs and an additional province (Madrid) with divergent behavior are observed. Provinces with developed agricultural and industrial economic sectors, high mobility, and a high proportion of Central and South American immigrants had the highest level of CI_14_. We show that the transmission of the virus is not homogeneous in the Spanish national territory. Our results are helpful for identifying differences in determinants that could explain the pandemic’s evolution and for formulating hypotheses about the effectiveness of implemented measures.

## 1. Introduction

The severe acute respiratory syndrome coronavirus 2 (SARS-CoV-2) novel betacoronavirus [1] may cause atypical pneumonia, severe acute respiratory syndrome, and a wide spectrum of clinical manifestations of different severity commonly called coronavirus disease 2019 (COVID-19) [2]. The worldwide spread and expansion of SARS-CoV-2 caused the COVID-19 pandemic with increased crude mortality rates in most European countries in 2020 [3]. SARS-CoV-2 infection is transmitted mainly by inhalation of respiratory droplets and aerosols emitted by a patient from the upper and lower respiratory tract or by direct contact with the conjunctiva of exposed individuals [4,5]. The SARS-CoV-2 transmission risk scales positively with the duration of exposure and the closeness of social interactions, with the highest per-contact risk estimated to be in households [6]; indoor transmission is very high compared to outdoors [7].

Spain is one of the countries that has suffered most from the COVID-19 pandemic [8]. In mid-March 2020, the Spanish government implemented a general lockdown period with a stay-at-home requirement that reduced community transmission [9].

SARS-CoV-2 infection can be detected in presymptomatic stages, be asymptomatic or paucisymptomatic (presenting few symptoms, extremely mild symptoms, or not very expressive symptoms). In Spain, 33% of the SARS-CoV-2 infected individuals were asymptomatic in spring 2020 [10]. SARS-CoV-2 RNA shedding can be prolonged; viral RNA detection may not correlate with the shedding of viable virus and infectivity [11]. Early diagnosis of infected individuals, including asymptomatic ones, is essential to ensure rapid and appropriate healthcare delivery and prevent further infection. Reverse transcriptase–polymerase chain reaction (RT–PCR) testing is the main diagnostic procedure for detecting SARS-CoV-2 infection [12]; in Spain, the rapid antigen detection test has been used as a valid diagnostic method since 22 September 2020 [13].

Asymptomatic SARS-CoV-2 infections play a substantial role in viral transmission [14,15,16], and this constitutes a challenging situation for the proper identification and tracking of any exposed contacts. The implementation of effective prevention and control strategies that target close contacts of infected individuals and the population at risk of poorer outcomes could help us to control the pandemic better. Quarantine for close contacts reduces transmission in undetected cases. Other strategies that have been implemented to date include physical, social distancing (avoidance or reduction of contacts, reduction of contact time, safety distance) and using protective elements, such as face masks [17]. Governments are applying restrictions on gatherings of people who do not live together, especially in closed spaces due to the high transmission rate in poorly ventilated areas [7,18]. These restrictions are being complemented by other restrictions on mobility, both internal and external.

Europe has experienced a second wave of the COVID-19 pandemic [19]. Strict social distancing measures have proven effective in curbing the incidence of COVID-19, but as restrictions were lifted, emerging second-wave scenarios [20] forced restrictive measures to be reimposed to prevent the collapse of the healthcare system. This second wave in Spain had its origins in summer 2020 when a variant of SARS-CoV-2 emerged and spread to other European countries [21,22], with the highest incidence among young adults [23].

Since the increase in infected individuals in autumn 2020 [24], Spain seems to have chosen a mitigation strategy [25]. The country has experienced cycles of escalation and de-escalation at a regional and local level with measures that weaken the economy to improve health protection while awaiting the vaccination of vulnerable groups.

Differences in population density, cultural behavior, population age structure, underlying comorbidity rates, and contact rates across groups influence transmission dynamics within communities [26], so the transmission can be heterogeneous. Further, the variation in transmissibility between individuals may play a major role in spreading SARS-CoV-2 [26].

Various studies have assessed determinants of the evolution of the COVID-19 pandemic [27] and examined the spatial patterns and underlying risk factors [28,29,30,31,32,33]. In the United States of America, income inequality, the percentage of nurse practitioners, the percentage of the black female population [29], county-level socioeconomic factors [30] and other social determinants, such as age, disability, language, ethnicity, occupation and urban status [28] explain significant variations in COVID-19 incidence. Urban areas, as well as territories with a high proportion of black individuals, have a significantly higher number of COVID-19 cases and mortality rates [28].

These determinants have not been thoroughly studied in Spain, a country with much geographical and cultural variability. Moreover, the Spanish mitigation strategy has been approached with a degree of territorial heterogeneity. This study assesses the territorial heterogeneity of the evolution of SARS-CoV-2 infection in Spain. The aim is to understand the spatial determinants of infections by comparing the variables described for SARS-COV-2 infection cases in the second wave of the pandemic in Spain.

## 2. Data and Methods

### 2.1. Database

This study is a time-series analysis using information from the Escovid19data collection [34]. This daily database was the only public access repository, including provincial information, when the present study was conducted (January 2021); it is a collaboratively developed database compiling data provided by the Spanish public administrations. For the present study, we used data for each Spanish province from 20 June 2020, when a complete data set for all provinces first became available, to 30 November 2020, before the adoption on 2 December 2020 of new specific measures in anticipation of the Christmas holiday celebrations [35].

### 2.2. Disaggregated Data from Spanish Provinces and Self-Governing Cities

Spain is a decentralized compound national state currently divided into 19 self-governing territories: 17 self-governing regions, each comprising one or more 19th century-implanted provinces (with a total of 50), and two self-governing cities (Ceuta and Melilla). The 1978 Spanish Constitution recognizes and guarantees self-government for the territories. The vast majority of the Spanish national territory has acceded to self-government until 1995. Other Spanish territories are directly government-run uninhabited small islands along the Strait of Gibraltar and the Southern Alboran Sea off the North African coast and an island bordering Morocco. All self-governing regions are named *comunidades autónomas* in Spanish, except the Chartered Community of Navarre (*Comunidad Foral de Navarra* in Spanish), whose own self-government regime is based on an age-old chartered codified consuetudinary law system granting or acknowledging rights and freedoms, the Act of Confirmation of Charters of 1839 and the Compromise Act of 1841. Canarias was divided in 1927 into two provinces: Santa Cruz de Tenerife and Las Palmas. Since then, only small changes have been made in provincial organization.

Public health competencies are the responsibility of the self-governing regions, except for those aspects of coordination between the regions, basic national criteria, and the management of supra-regional alerts, which correspond to the national level (the Spanish Parliament and Spanish Department of Health). Public health competencies remain at the national level for the self-governing cities of Ceuta and Melilla. The Interterritorial Board of the Spanish National Health System is the permanent body for coordination, cooperation, communication and information between the self-governing regions’s health departments, the self-governing cities’s health departments, and the Spanish Department of Health, providing cohesion to the system. In case of special risk for public health, with the prior agreement of the Interterritorial Board of the Spanish National Health System, the declaration of coordinated actions in public health by the Spanish Department of Health obliges all the parties to comply. These coordinated actions include, among others, the strengthening of epidemiological information systems for decision-making and health promotion and implementing disease prevention and control programs when the risk transcends the regional sphere [36].

To face the second wave of SARS-CoV-2, the Spanish government declared in October 2020 a new state of alarm throughout the national territory, by Royal Decree 926/2020 [37], which was extended until May 2021 by Royal Decree 956/2020 [38]. This state of alarm established a nationwide curfew, except in Canarias, and delegated to the authority of each self-governing region or self-governing city the adoption of specific measures to limit mobility and assembly in the corresponding territory according to socio-epidemiological indicators. Under Royal Decree 926/2020, the competent authority was the Spanish government during the state of alarm, and, in each territory, the delegated competent authority was the regional government. The delegated competent authorities was empowered to dictate, by the delegation of the Spanish government, decrees, orders and subordinate instruments for establishing regulations or ad hoc decisions concerning limitations, constraints or restrictions of circulation, mobility and assembly. Although decision-making on public health measures continued to be carried out at the regional level, we have used data from the provinces and self-governing cities to use the most disaggregated data available. Both single and multiprovincial regions can take measures at a lower level when they deem it necessary. To guarantee the required coordination in applying the measures contemplated in the Royal Decree 926/2020, the Interterritorial Board of the Spanish National Health System may adopt for these purposes, in addition to coordinated actions, as many agreements as it considers necessary, including when applicable, establishing reference indicators and risk assessment criteria [37].

To face the second wave of SARS-CoV-2, the Spanish government declared in October 2020 a new state of alarm throughout the national territory, by Royal Decree 926/2020 [37], which was extended until May 2021 by Royal Decree 956/2020 [38]. This state of alarm established a nationwide curfew, except in Canarias, and delegated to the authority of each self-governing region or self-governing city the adoption of specific measures to limit mobility and assembly in the corresponding territory according to socio-epidemiological indicators. Under Royal Decree 926/2020, the competent authority was the Spanish government during the state of alarm, and, in each territory, the delegated competent authority was the regional government. The delegated competent authorities was empowered to dictate, by the delegation of the Spanish government, decrees, orders and subordinate instruments for establishing regulations or ad hoc decisions concerning limitations, constraints or restrictions of circulation, mobility and assembly. Although decision-making on public health measures continues to be carried out at the regional level, we have used data from the provinces and self-governing cities to use the most disaggregated data available. Both single and multiprovincial regions can take measures at a lower level when they deem it necessary. To guarantee the required coordination in applying the measures contemplated in the Royal Decree 926/2020, the Interterritorial Board of the Spanish National Health System may adopt for these purposes, in addition to coordinated actions, as many agreements as it considers necessary, including when applicable, establishing reference indicators and risk assessment criteria [37].

### 2.3. Variables

To measure the evolution of the COVID-19 pandemic in the Spanish provinces and self-governing cities, we used the cumulative incidence for 14 days (CI_14_), defined as the total number of newly notified confirmed SARS-CoV-2 infections per 100,000 inhabitants over the previous 14 days. Given that some Spanish provinces have less than 100,000 inhabitants, we have also considered the notified confirmed SARS-CoV-2 infections per 10,000 inhabitants in the last 14 days. The results obtained hardly vary concerning those presented here and are available upon request. We have decided to use CI_14_ because this is the rate employed to take health decisions. Cumulative incidence is the proportion of healthy individuals who develop the disease over a given period. The CI_14_ series allows an exhaustive assessment of transmission speed and constitutes a powerful epidemiological tool to assess how the COVID-19 pandemic evolves; lower values suggest a better control of the pandemic. Notified confirmed SARS-CoV-2 infections included RT–PCR-positive and antigen-positive cases [39,40,41,42]. Reinfections were not assessed in the period under study.

The Interterritorial Board agreed at the end of September to aim to achieve CI_14_ below 60/100,000 inhabitants. It established that cities of more than 100,000 inhabitants that had a CI_14_ of more than 500 cases, a positivity of 10%, and an ICU bed occupancy greater than 35% would take extraordinary measures [43]. Among others, these indicators would categorize the provinces in terms of their alert level [44]. The Council of the European Union established a CI_14_ threshold of 25 cases per 100,000 inhabitants to indicate increasing risk [45]. Therefore we used the main indicator employed by the Spanish and European authorities (CI_14_).

The explanatory variables (x_i_) for ordered logit methods were selected, taking into account the results of Turner-Musa et al. (2020) [27] and Andersen et al. (2021) [28]. These studies report on the determinants for the evolution of COVID-19 in the USA and consider data availability. Following these authors, we have chosen variables, including mobility, population density, economic structure, income, education, ethnicity, age, health status, healthcare, and seroprevalence in November 2020, global seroprevalence until November 2020 and the test positivity rate in November 2020. The list of the considered variables is presented in Appendix A with the sources from which they were obtained.

### 2.4. Convergence and Phillips–Sul Methodology

The concept of convergence has a great tradition in economic literature. Many articles have been discussed, with per capita gross domestic product (GDP) being the most commonly used economic indicator. Following the seminal paper by Barro and Sala-i-Martí (1992) [46], we can conclude in favor of convergence between the per capita GDP of a group of countries whenever the dispersion of the per capita GDP values reduces across the sample. Consequently, if convergence exists, then the cross-section variance of the per capita GDP goes to 0. This type of convergence is commonly known as σ-convergence.

We have recently observed increased this type of analysis, mostly due to the contributions of Phillips and Sul (2007, 2009) [47,48] (hereafter PS), who designed a very popular statistic that has been extensively employed to test for convergence. Additionally, as we see below, this methodology also allows the researcher to analyze whether the evolution of the variables is becoming similar. Although these studies were initially focused on macroeconomic indicators [49,50,51], this interest has extended to non-economic variables, including health indicators. Examples are the papers by Duncan and Toledo (2020) [52], Kasman and Kasman (2020) [53], Christopoulos and Eleftheriou (2020) [54] or González-Álvarez et al. (2020) [55], among others.

Following PS, let us consider that *X_it_* represents the log of the health indicator of interest, the CI_14_ in this particular case, with *i* = 1, 2,…, 52 (the 50 Spanish provinces and 2 self-governing cities) and *t* goes from 20 June to 30 November 2020. This variable can be decomposed as *X_it_* = *δ_it_μ_t_*, where *μ_t_* and *δ_it_* are the common and the idiosyncratic components, respectively. PS suggest testing for convergence by analyzing whether *δ_it_* converges towards *δ*. To do so, they first define the relative transition component:

(1) $$h_{it}=\frac{X_{it}}{N^{-1}\sum_{i=1}^{N}X_{it}}=\frac{\delta_{it}}{N^{-1}\sum_{i=1}^{N}\delta_{it}}$$

In the presence of convergence, *h_it_* should converge towards unity, while its cross-sectional variation, *H_it_*, is defined as follows:

(2) $$H_{it}=N^{-1}\overset{N}{\underset{i=1}{\sum }}{\left(h_{it}-1\right)}^{2}\overset{As}{\to }0,\;as\;T\overset{as}{\to }\;\infty$$

and should go to 0 when *T* goes towards infinity. Then, PS test for convergence by estimating the following equation:

(3) $$log\frac{H_{1}}{H_{t}}-2log\left[\log\left(t\right)\right]=\alpha +\beta \log\left(t\right)+u_{t},\;t=T_{o},\;\ldots ,\;T$$

with *T_0_* = [*rT*]. PS recommend using the value r = 0.3. Equation (3) is commonly known as the log-t regression. The null hypothesis of convergence is rejected whenever the parameter is lower than 0. PS suggest estimating model (3) by methods that correct for the presence of autocorrelation and heteroskedasticity (HAC methods) and, later, employ the *t*-statistic to test the null hypothesis *β* = 0. The use of these robust methods ensures that this *t*-ratio converges towards a standard *N*(0,1) distribution, and, therefore, we will reject the null hypothesis of convergence whenever this *t*-statistic takes values lower than −1.65.

PS warn of the possible presence of artificial convergence. An obvious example of this is the case of the consumer price index. If the base year is taken at the end of the sample, then these indicators would seem to converge, given that the base year takes the value 100 for all the cross-section units. To avoid such artificial forms of convergence, PS suggest taking the first observation as the base year, rescaling the data and discarding some initial observations to avoid the effect of the new initial observation. In the present case, the effective sample for the PS analysis begins on 1 September 2020.

If we reject convergence, PS propose the following robust clustering algorithm for identifying clubs in a panel:

- Order the *N* provinces according to their final values;
- Starting from the highest-order province, add adjacent provinces from our ordered list and estimate model (3). Then, select the core group by maximizing the value of the convergence *t*-statistic, subject to the restriction that it is greater than −1.65;
- Continue adding one province at a time of the remaining provinces to the core group, and reestimate model (3) for each formation. Use the sign criterion (*t*-statistic >0) to decide whether a state should join the core group;
- For the remaining provinces, repeat steps (ii)–(iii) iteratively and stop when clubs can no longer be formed. If the last group does not have a convergence pattern, conclude that its members diverge.

PS recommend performing club merging tests after running the algorithm using Equation (3) to avoid an overestimation of the number of clubs.

Finally, we have followed the suggestion of PS and extracted the trend components of the series by filtering them using the Hodrick and Prescott (1997) filter [56]. The value of the parameter λ has been calculated according to the results of Ravn and Uhlig (2002) [57], who recommend employing the rule λ = 1600 p^4^, with p being the number of periods per quarter (365/4). To study the forces that may drive creating these clubs, we have estimated the following model:

*y_i_* = *x_i_*′ *β* + *u_i_* (*i* = 1, 2,…, 52) (4)

The dependent variable *y_i_* may have various possible outcomes, each of them related to the number of clubs that the PS methodology has estimated. Then, *y_i_* = *j*, if the *i*-th province is included in the *j*-th club, with *i* = 1, 2,…, 52 and *j* = 1, 2,…, *J*, with J being the number of estimated convergence clubs. These different *J* values imply a preference or ordering of the clubs, which should be considered in the estimation. Therefore, ordered logit methods should be employed with the chosen explanatory variables (*x_i_*).

## 3. Results

Our data shows that CI_14_ varied significantly across provinces from June to November 2020, identifying different behavior patterns. A brief descriptive analysis is included in Table 1.

The results confirm the heterogeneous behavior of the CI_14_ for the Spanish provinces. If we consider the initial CI_14_ values, most provinces show values lower than 50, the exceptions being Soria, Lérida and Madrid. The CI_14_ values at the end of the sample are even more different. A few provinces show CI_14_ values lower than 150: Las Palmas, Orense, and Santa Cruz de Tenerife, and the Madrid CI_14_ are close to this value. The rest exhibit much higher values; 38 provinces have values higher than 250, and 6 provinces show CI_14_ values higher than 500. The worst CI_14_ values are those of Palencia, Valladolid and Burgos, all of them located in the inner region of Castilla y León. The minimum and, especially, the maximum values also reveal a large degree of heterogeneity. The minimum values occur at the beginning of the sample, as expected, and their standard deviation is relatively small (11.13). The opposite occurs with the highest values. To appreciate their heterogeneity, we should note that the standard deviation is 329. The lowest maximum CI_14_ value is 125.1 (Santa Cruz de Tenerife). The rest of the maximum values exceed 300, and more than half of the provinces exhibit maximum values greater than 700. The worst values are those of Huesca, Melilla, and Granada. However, despite this heterogeneity, we should note that the variation coefficient of the CI_14_ values is relatively low, with just 17 provinces showing a value greater than 1, which indicates that the CI_14_ has not varied very much among the provinces; the highest value was observed in Huelva (138%).

**Figure 1:**
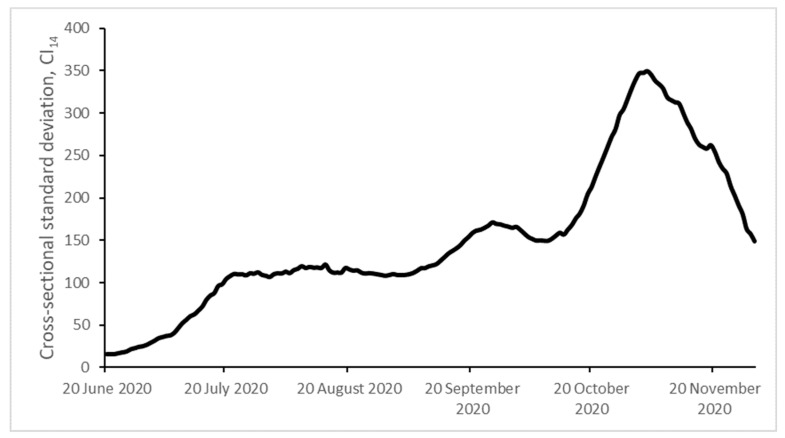
σ-Convergence. Cross-sectional standard deviation.

**Figure 2:**
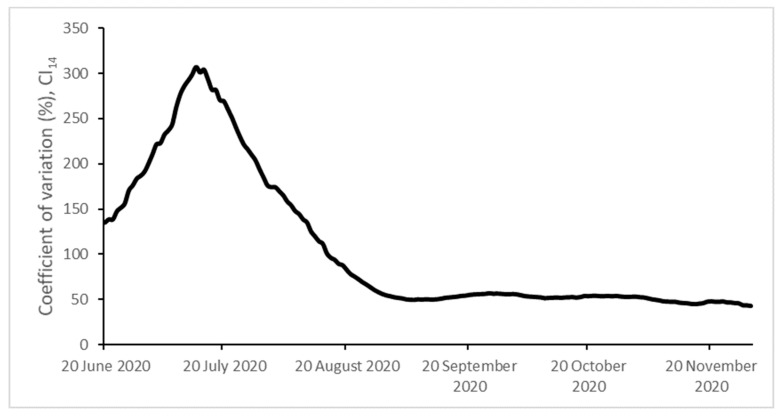
σ-Convergence. Coefficient of variation.

We aim to analyze the evolution of the Spanish provincial CI_14_ using the PS methodology. The results of its application are presented in Table 2. As we can see, the null hypothesis of convergence is rejected. This result is also supported by Figure 1 and Figure 2, showing that the cross-sectional dispersion does not tend towards 0 and offering additional evidence of the absence of σ-convergence. Then, we reject the presence of a single pattern of behavior in the evolution of the CI_14_ across the Spanish provinces and self-governing cities.

**Figure 3:**
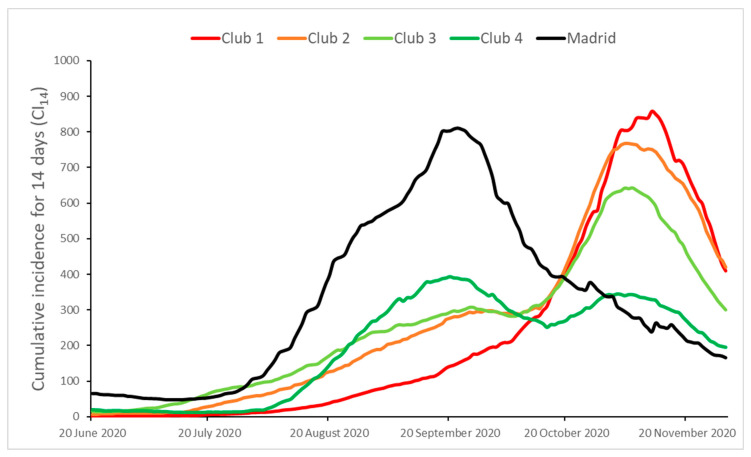
Average values of the estimated clubs.

**Figure 4:**
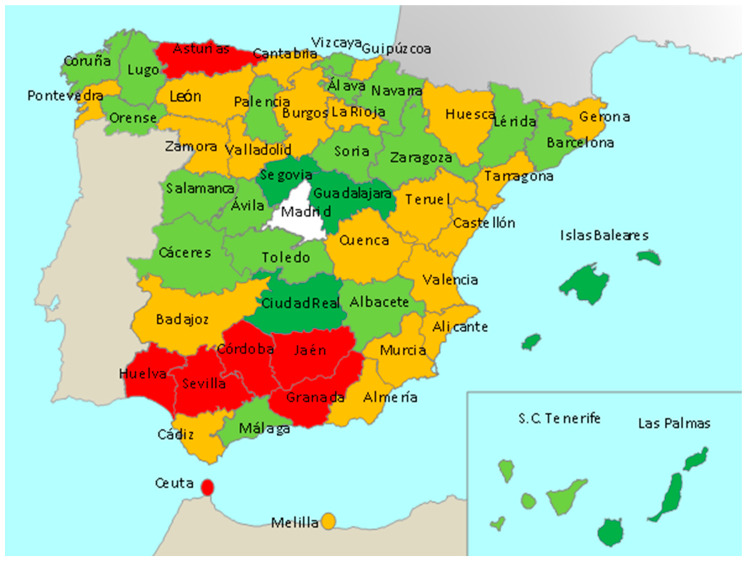
Geographical representation of estimated clubs in a map of Spanish provinces. The clubs are shown in different colors: Club 1 in red, club 2 in orange, club 3 in light green, club 4 in dark green, and the divergent behavior province (Madrid) in white.

Table 2 shows the presence of some convergence clubs. The use of the PS cluster algorithm allows us to identify four statistically different convergence clubs and an additional province (Madrid) in the sense that the evolution of its CI_14_ is not similar to any of the estimated clubs. To better understand the behavior of the estimated clubs, Figure 3 presents the corresponding average CI_14_ values, and Figure 4 represents the distribution of the convergence clubs in a map of the Spanish provinces. Club 1 is formed by Asturias (a uniprovincial region), the city of Ceuta and a compact set of 5 southern provinces within the self-governing region of Andalucía. Mediterranean coast provinces are mostly included in club 2 (Barcelona, Málaga, and Granada are exceptions). A few inner provinces are also included in club 2: Burgos, La Rioja, Valladolid, León, Zamora, Badajoz, Huesca, Teruel, and Cuenca. The north coastal provinces exhibit a quite heterogeneous behavior, with Asturias included in club 1, Cantabria, Guizpúzcoa and Pontevedra in club 2, and the rest in club 3. Finally, the island provinces are included in clubs 3 (Santa Cruz de Tenerife) and 4 (Islas Baleares and Las Palmas) and the rest of the inner provinces.

The evolution of the average CI_14_ values of the estimated clubs is presented in Figure 3, allowing us to appreciate the cross-section differences and providing some additional insights. The club 1 and club 2 behaviors were quite similar and exhibited the highest values at the end of the sample. However, they differ in their evolution during the summer months and the beginning of autumn. The club 2 CI_14_ average values were always greater up to the beginning of November. After this, club 1 attains its maximum (858) on 11 November 2020, while the club 2 maximum was a little lower (768) and occurred slightly earlier (5 November). From then onwards, both of them showed a sharp drop, and the values at the end of the sample were similar (410 and 420).

Club 3 is quite closely related to club 2, even exhibiting greater CI_14_ values up to the end of September. Later, club 3 moderated its growth compared to club 2 (2.2 and 2.8, respectively) and reached its maximum on almost the same day (6 November), with a lower value (643). The decreasing growth rate up to the end of the sample was greater for club 3 (−3.3%) than for club 2 (−2.4%) and, therefore, the value of club 3 (300) was lower than the value of club 2.

The club 4 behavior was a combination of the patterns of behavior of Madrid (up to mid-October) and of clubs 1–3, although the evolution was smoother in club 4. Consequently, it shows an almost bi-modal evolution, with peaks on 20 September (393) and 2 November (344).

Finally, the Madrid province diverged from the rest of the clubs (Figure 3). Madrid shows the greatest CI_14_ up to mid-September. The maximum value (810) was similar to the maximum values of clubs 1 and 2, but it was attained one month and a half earlier (22 September).

For all the clubs, we observed that the higher the CI_14_ maximum in the first part of the study period, the lower it is during the second part. As we have seen in the territorial analysis, the coastal provinces (except the islands) exhibited a worse performance than the rest of the provinces in terms of CI_14_.

We have estimated an ordered logit model to determine which forces may have generated our estimated clubs. These results are presented in Table 3. The final specification was obtained as follows: We have first run a forward selection procedure using ordinary least-squares. Later, we have estimated the ordered logit model by nonlinear maximum-likelihood methods, removing those explanatory variables that were not significant at 5%. It seems sensible to mention that, even though the sample size is relatively small, the maximum-likelihood estimator works properly, and the nonlinear algorithm achieves convergence in very few iterations. The Appendix A includes a list of the initial variables, as well as their average values for each of the estimated convergence clubs.

We should note that we have employed the statistic proposed by Brant (1990) [58] to test the assumption of the proportionality of the odds ratios. As we can see, we cannot reject this null hypothesis and using the ordered logit model seems to be appropriate. We should also mention that Table 3 includes estimating the coefficients of the model (4), with the estimated cluster robust standard errors in parenthesis. The explanatory power of the estimated model is relatively high, being able to correctly classify 69% of the provinces.

Our results show that mobility—measured by the number of travelers—, the economic structure—especially the percentage of working people devoted to the agriculture and industry sectors—, the health status—measured by life expectancy at birth—, and the number of Central and South American immigrants are the variables included in the final specification of the estimated logit model. This final model also includes the variable capital of the region, which takes the value 1 if the province contains the region’s capital and 0 otherwise. The higher the mobility towards the province and the percentage of people working in the two above-mentioned sectors, the more probable the province is included in clubs 1 and 2, which have the highest levels of CI_14_. Similarly, the probability of being included in these clubs is greater for the provinces that include the region’s capital. By contrast, the better the health status previous to the pandemic, the more probable the province is included in clubs 3 and 4, the ones with the lowest CI_14_ values.

To facilitate the interpretation of the impact of the explanatory variables on the probability of membership in a specific club, we have included in Table 3 the resulting marginal effects, all of them computed at the mean of all the explanatory variables. The marginal effects show the change in the probability of belonging to a specific club given a small change in the explanatory variables. We can see that the variables of the number of Central and South American immigrants and life expectancy at birth have the largest impact.

## 4. Discussion

This study shows that the evolution of the COVID-19 pandemic has been quite heterogeneous across the Spanish provinces, revealing several patterns of behavior. This is in line with other observations in larger countries [28,29]. Through an ordered logit model, we have analyzed some variables that may influence the temporal and territorial evolution of the COVID-19 pandemic, such as mobility, economic structure, immigration, and life expectancy at birth.

In terms of the number of travelers to the province, mobility is associated with developing the provincial incidence of the infection. We can also appreciate that those provinces containing the capital of a region also exhibit higher incidence levels, clearly connected with this mobility factor. The clubs with the highest mobility are club 4 and club 2; club 2, together with club 1, have the worst SARS-COV-2 evolution. However, club 4 is the most benevolent in terms of CI_14_, which may be related to the islands and transit-to-Madrid travels. These associations should be studied more in-depth [59,60]. Internal mobility restrictions proved effective in reducing SARS-CoV-2 transmission in the first wave [61], but they have not been evaluated territorially in Spain in the succeeding phases in a different way than a stay-at-home requirement. A recent study reported that reducing mobility positively affects reducing the growth rate of incidence [62]. Tools to assess spatial mobility in real time would help in the design and implementation of more effective local-targeted interventions [59]. Another study has shown that a more effective way to reduce CI_14_ could be to limit the accumulation of people in specific places. A small minority of superspreader events account for many infections, and measures to restrict the maximum occupancy of spaces are more effective than uniformly reducing mobility [63]. Studies on SARS-CoV-2 superspreading events suggest that heterogeneity in infectivity may have a significant impact on transmission dynamics [64], calling for further research on factors that could influence interindividual heterogeneity, susceptibility, and clinical outcome [26]. The provincial demographic structure—population density and population in cities lower than 50,000—could be related to the SARS-CoV-2 transmission and CI_14_. Club 1 shows a population density much higher than the rest of the clubs (721) and a proportion of the resident population in municipalities of less than 50,000, which takes the value 4.0, very different from the rest of the clubs: >12.6 (Appendix A). Further studies are necessary to identify and understand associations between mobility, superspreader events, demographic structure, and social interaction [65].

The differences observed between club 1 and the rest of the clubs suggest that provinces with high agricultural employment could be more vulnerable to SARS-CoV-2 infection. The population of the industrial sector is another determining factor of high CI_14_. The highest proportion of industrial activity is related to club 2, which includes the highly industrialized provinces on the Mediterranean coast. The population, which works in the industry, would have more significant opportunities for person-to-person interaction. By contrast, the construction sector (Appendix A), which is more dominant in clubs 2–4, could probably be more protected against high SARS-CoV-2 incidence. Further, the population with the most extensive service sector (club 4) did not have higher CI_14_ than the rest of the clubs, so it could be assumed that this sector has adapted to SARS-CoV-2 prevention measures [66]; these provinces include the islands, whose economy relies on the service sector.

Among the immigrant groups considered, those from Central and South America have shown a correlation with the SARS-CoV-2 CI_14_. The percentage of Central and South American immigrants helps us to discriminate between CI_14_ clubs. However, this factor should be interpreted with caution. While helping us to understand the estimated model results, it does not imply causality. We have no evidence to determine whether ethnic variables help us to explain spreading this pandemic, considering that Andersen et al. (2021) [28] showed black individuals and non-English speakers were significant predictors of COVID-19 cases. Moreover, the percentage of immigrants can be a proxy for wealth and economic development and social interaction [67], although there is no association with per capita GDP or the Human Development Index (HDI).

Spain has one of the highest life expectancies at birth estimations in the world [68]. This variable, as well as the infant mortality rate, is commonly employed to reflect the global status of a population [69,70]. It is true that there are other options and that alternative measures, such as the quality-adjusted life-year (QALY), are also of great interest. Unfortunately, data availability obliges us to focus our study on the former variables. The data in Appendix A show that the slightly lower life expectancy at birth observed in club 1 (Appendix A), following recently published results that show lower baseline life expectancy estimations in Andalucía (along with most provinces in club 1) [71], could be associated with the higher transmission of the virus.

Seroprevalence at a particular moment—the proportion of the population with immunity—is related to the cumulative incidences of each province until that point in time. Even so, high seroprevalence levels may reduce the circulation of the virus due to the higher proportion of non-susceptible individuals. Seroprevalence in Spain is far from reaching the herd immunity threshold for COVID-19 (~82.5%), using the mathematical formula 1–1/R0 and assuming an R0 estimate of 5.7 [72,73]. The herd immunity threshold would mean that the incidence of infection will begin to decline once the proportion of individuals with acquired immunity to SARS-CoV-2 in the population exceeds 82.5%. It has been shown that in the second half of November, 7.1% of the population residing in Spain had anti-SARS-CoV-2 immunoglobulin G (IgG) antibodies, without significant differences between sexes (CI95: 6.7–7.6; 7.5% in women versus 6.7% in men) [74]. In the territorial disaggregation, seroprevalences higher than 10% are found in the provinces of Ávila, Segovia, Soria, Palencia and Salamanca (Castilla y León), and Lérida (region of Cataluña). These provinces do not correspond to any CI_14_ cluster, but there are differences in the seroprevalence values in November for each CI_14_ club: clubs 1 and 2 had on average seroprevalences lower than 6.2%, and clubs 3 and 4 greater than 7.4%; in Madrid figure was 12.5%. IgG antibodies are only part of the anamnestic immune response, and memory cells play a fundamental role in adaptive cellular immunity; development of memory B and T cells is critical for long-term protection [75,76]. Although the serum levels of anti-SARS-CoV-2 IgG antibodies show a decline at months 6–8, virus-specific T and/or memory B cell responses increase with time and are maintained during at least 6–8 months after infection [76,77,78,79]. Asymptomatic infected patients generate a weaker immune response [80], and some COVID-19 patients experience a decline in B cell responses over a timescale of 3 months [81,82,83], so T cell responses may be more sensitive indicators for SARS-CoV-2 infection prevalence than seropositivity [84]. Nevertheless, the seroprevalence results (Appendix A) in November 2020 correspond to the immunity generated by infection during the analyzed previous period. Conversely, the IgG seroprevalence results in November 2020 underestimate the past infection of more than six months.

The global seroprevalence—the percentage of people in the population with IgG antibodies against the SARS-CoV-2 virus at any time since the beginning of the study—enables us to better estimate the infection rate since the beginning of the pandemic, considering that long-term immunity remains uncertain [85]. The Spanish global seroprevalence in November was 9.9% (95% CI: 9.4–10.4) [74]. A marked geographical variability in global seroprevalence was observed: only Las Palmas, Santa Cruz de Tenerife, La Coruña, Pontevedra, Lugo, Valencia, Huelva and Córdoba presented accumulated prevalences below or close to 5%; the central provinces around Madrid showed more than 15% (Cuenca, Soria and Madrid more than 18%). Above or around 10% is the entire central nucleus of the country. This does not correspond to the CI_14_ patterns, but club 1 and club 2 had seroprevalences lower than 8.7%, and club 3 and club 4 greater than 11.1%; the Madrid global seroprevalence was 18.6%. Furthermore, this global seroprevalence could correspond to the underdetected first wave incidence, whose territorial differences have not been assessed in this article. SARS-CoV-2-reactive T cells are associated with protection from symptomatic SARS-CoV-2 infections, as reported in a prospective cohort study [86], and it has been suggested that seropositivity could be associated with protection from infection [84,87]. Coronavirus protective immunity could be short-lived, and it has been suggested that it provides no long-term protection from reinfections; reinfections occurred most frequently at 12 months after infection [88].

Among the variables relating to detection systems for SARS-COV-2 infection, we have considered the test positivity rate (TPR), whose data from 24–30 November 2020 were accessible [89]. As the groups had a worse evolution at the end of the period (club 1 and club 2), TPR was higher (>10.8), suggesting that the diagnostic capacity could not detect the full burden of infection and that more testing should probably be done. High TPRs also suggest high CI_14_ due to high transmission in the community. Low TPRs not only shows a good control; it can be influenced by an excessive consideration of close contacts without real risk or by screening or studies in populations with low prevalences. The better clubs (club 3 and club 4) had a TPR > 7.5, above recommendations for getting transmission under control.

Madrid and club 4, which had the lowest CI_14_ at 24–30 November 2020, also had the lowest TPR (7.5–7.6). This assessment was a specific one and, therefore, is not representative of the entire series. In Madrid, the transmission was difficult to control at the pandemic’s peak on 22 September when it had a TPR of 23.0%, higher than the other clubs [90].

Other variables that may influence the SARS-CoV-2 CI_14_ evolution are the underdiagnosis and delay in notification of some regions, the number of tests carried out per 100,000 inhabitants, the average test positivity rate in close contacts, and the average number of close contacts in confirmed cases.

To the best of our knowledge, the present study is one of the first studies to demonstrate the existence of different patterns of the COVID-19 pandemic evolution in Spain. These results could help identify specific differences between geographical areas that could be potential factors in planning for better outcomes. Such differences may be related to the population’s socio-epidemiological characteristics. The restriction measures adopted different health policies, etc. This could help us to formulate various hypotheses and assess the efficacy of specific measures and policies [91].

Decision-making in good time and at the territorially disaggregated level allows decisions to be more easily adapted to the epidemiological situation and to the risk in specific geographical areas and it limits and circumscribes the effects on the economy of specific restriction measures aimed at reducing social interaction between non-cohabiting people, such as restrictions on economic and commercial activity, and territorial mobility. Disaggregation in decision-making seems to be a necessary solution given the foreseeable persistence of SARS-CoV-2 transmission in the coming months until complete vaccine coverage reduces transmission and the number of infected people.

The evolution of the pandemic depends on citizens’ behavior in terms of social interaction. Public authorities have controlled mobility to limit social interaction [59]. Lockdown interventions increase transmission risk within families and households, whereas the timely isolation of infected individuals reduces risk across all types of contacts [6]. The high incidence rates of SARS-CoV-2 infection observed during autumn 2020 led to a state of an alarm first in Madrid in October (Royal Decree 900/2020 [92]), applied in Madrid’s descending epidemic phase, and approximately two weeks later in the whole country (Royal Decree 926/2020 [37]). Since then, many regions and cities have maintained high mobility restrictions and economic and commercial activity limitations, with significant differences in the adopted measures. Other variables in terms of security measures could be related to cultural factors within the population’s variability not yet analyzed in Spain [93].

The main strength of our study is that we employ time-series techniques, which are quite useful for providing time-consistent results. Furthermore, the database includes all notified confirmed SARS-CoV-2 cases in Spain during the study period. This study has focused on the evolution of SARS-CoV-2 infection, but information on other important variables has not been used, for example, the patient’s health status, clinical severity, mortality, case-fatality rates, infection fatality rates or other sociodemographic characteristics. The most serious limitation of this paper is the quality of the data, an aspect that should be improved in the future by the Spanish authorities and that would lead to more robust research. We should also recall that our results do not offer evidence of causality, a question that is left to future research.

In this regard, we consider that further research is needed to explain the determinants of the differences between geographical areas and pandemic behavior in the future. We aim to study various factors that could contribute to different patterns across Spain, including health policies, results from vaccination programs, and restriction measures. It is crucial to consider the nature of the measures implemented in different regions and cities to correctly interpret changes in the pandemic’s impact. This could also help us to identify the most effective measures.

## 5. Conclusions

This study shows that the transmission of the SARS-CoV-2 virus is not homogeneous in the Spanish national territory. We performed an exploratory analysis comparing geographical areas to form hypotheses regarding specific factors associated with poorer control of the pandemic, taking into account the population characteristics, health policies, and the measures adopted.

Our results show that mobility, economic structure, migration and overall provincial health status are strongly associated with COVID-19 outcomes. However, we recognize that additional studies are necessary to study in detail the causality of the variables that we have included in our model.

## Figures and Tables

**Table 1:** Descriptive analysis.

	Initial	Final	Max	Min	CV (%)
Alacant/Alicante	1.1	358.8	394.2	1.0	91.3
Albacete	8.8	330.3	510.1	7.0	77.2
Almería	0.8	379.2	482.8	0.7	72.4
Araba-Álava	9.2	370.4	545.9	8.7	66.7
Asturias	0.8	398.0	649.7	0.1	123.5
Ávila	19.0	241.7	783.4	1.9	88.6
Badajoz	1.5	205.9	700.5	1.5	99.2
Balears, Illes/Baleares, Islas	5.4	185.6	335.8	4.6	72.0
Barcelona	11.5	234.9	809.0	9.6	83.3
Bizkaia/Vizcaya	23.5	367.2	745.5	3.1	73.7
Burgos	9.0	759.5	1387.8	3.4	97.8
Cáceres	5.1	260.8	508.7	2.3	87.0
Cádiz	2.2	524.0	579.9	1.2	112.2
Cantabria	3.6	338.0	547.4	1.9	91.9
Castelló/Castellón	3.3	309.3	485.0	2.2	103.4
Ceuta	0.1	218.2	838.7	0.1	116.0
Ciudad Real	44.6	297.1	510.9	19.6	76.0
Córdoba	0.3	296.9	776.5	0.1	104.3
Coruña, A/Coruña, La	4.0	224.0	349.8	2.9	82.1
Cuenca	25.0	520.6	1000.4	2.0	103.1
Girona/Gerona	11.9	299.7	964.5	8.0	102.7
Gipuzkoa/Guipúzcoa	3.5	569.9	1103.5	3.5	96.6
Granada	2.1	555.3	1485.3	2.1	128.7
Guadalajara	29.9	233.5	659.5	11.6	76.2
Huelva	0.2	380.7	576.4	0.2	137.6
Huesca	21.8	368.8	1429.7	21.8	80.6
Jaén	1.7	480.3	1012.5	0.9	119.8
León	9.6	465.9	897.0	2.2	104.0
Lleida/Lérida	59.3	360.3	731.6	59.3	53.4
Lugo	2.7	240.3	310.1	1.2	77.7
Madrid	65.9	166.4	810.7	48.4	71.2
Málaga	2.5	252.6	368.1	2.3	77.6
Melilla	2.3	420.9	1469.6	2.3	110.7
Murcia	1.2	279.7	730.6	1.2	84.6
Navarra	8.1	286.3	1270.4	8.1	84.7
Ourense/Orense	2.3	120.9	477.2	0.3	99.3
Palencia	24.8	607.5	1033.0	5.0	96.7
Palmas, Las	2.2	38.5	313.5	1.7	109.6
Pontevedra	4.9	274.2	359.3	0.6	115.0
Rioja, La	3.5	435.2	798.3	1.6	82.5
Salamanca	18.8	296.0	1046.6	2.4	91.4
Santa Cruz de Tenerife	1.2	123.8	125.1	0.2	79.4
Segovia	19.6	221.4	510.7	10.4	73.6
Sevilla	0.7	349.2	798.6	0.2	115.8
Soria	57.5	496.4	854.1	27.1	77.2
Tarragona	2.2	189.0	868.8	0.4	105.4
Teruel	6.7	416.7	1153.3	6.0	78.6
Toledo	12.5	351.7	840.5	4.0	81.4
València/Valencia	4.8	379.5	454.3	3.5	85.5
Valladolid	17.3	743.1	1200.1	4.8	92.9
Zamora	6.4	587.7	1050.8	0.6	104.0
Zaragoza	7.3	317.3	1050.9	6.8	63.5

This table presents some descriptive statistics of the CI_14_ of the Spanish provinces and self-governing cities (Ceuta and Melilla) for the considered sample. The columns “Initial” and “Final” present the values at the beginning and at the end of the sample, respectively. The columns “Max” and “Min” are the maximum and the minimum values of the series. Column “CV” reflects the coefficient of variation. Official or co-official provincial names are placed first for better identification. When the province has an official/co-official name in a local language, the Spanish denomination is placed after it. In the case of a hyphenated name, the official name includes both the local and Spanish languages. For clarity, Spanish names are used in the rest of the paper.

**Table 2:** Testing for convergence and convergence clubs.

Panel I. Testing for Convergence						
	Provinces	PS				
	Full sample	−0.809 (−218.01)				
Panel II. Convergence Clubs						
**Panel A. Initial Estimation**			**Panel B. Adjacent Analysis**		**Panel C. Final Estimation**	
**Initial Clubs**	**Provinces**	**PS**	**Merging**	**PS**	**Final Clubs**	**Provinces**
C1	Asturias, Córdoba, Granada, Huelva, Jaén, Sevilla, Ceuta	0.211 (10.56)	C1 + C2	−0.46 (−412.57)	Club 1	Asturias, Córdoba, Granada, Huelva, Jaén, Sevilla, Ceuta
C2	Alicante, Almería, Badajoz, Burgos, Cádiz, Cantabria, Castellón, Cuenca, Gerona, Guipúzcoa, Huesca, León, Murcia, Pontevedra, La Rioja, Tarragona, Teruel, Valencia, Valladolid, Zamora, Melilla	0.019 (1.30)	C2 + C3	−0.1173 (−8.99)	Club 2	Alicante, Almería, Badajoz, Burgos, Cádiz, Cantabria, Castellón, Cuenca, Gerona, Guipúzcoa, Huesca, León, Murcia, Pontevedra, La Rioja, Tarragona, Teruel, Valencia, Valladolid, Zamora, Melilla
C3	Barcelona, Cáceres, La Coruña, Lérida, Lugo, Málaga, Navarra, Orense, Palencia, Santa Cruz de Tenerife, Zaragoza	0.173 (11.86)	C3 + C4	0.023 (1.63)	Club 3	Álava, Albacete, Ávila, Barcelona, Cáceres, La Coruña, Lérida, Lugo, Málaga, Navarra, Orense, Palencia, Salamanca, Santa Cruz de Tenerife, Soria, Toledo, Vizcaya, Zaragoza
C4	Álava, Albacete, Ávila, Salamanca, Soria, Toledo, Vizcaya	0.261 (17.40)	C4 + C5	0.145 (2.58)	Club 4	Islas Baleares, Ciudad Real, Guadalajara, Las Palmas, Segovia
C5	Islas Baleares, Las Palmas, Segovia	0.613 (2.77)	C5 + C6	0.445 (14.15)	Divergent	Madrid
C6	Ciudad Real, Guadalajara	0.327 (23.75)	C6 + Divergent	−1.819 (−40.13)		
Divergent	Madrid					

This table presents the results of the PS methodology. Panel I includes the analysis of the null hypothesis of convergence. The value in parentheses is the log-t ratio, and the value above it corresponds to the estimation of the parameter *β* in (3). The null hypothesis is rejected if the log-t ratio is lower than −1.65. For the sake of clarity, Spanish names are used for all provinces. Panel II presents the results of applying the PS clustering algorithm. Panel A shows the initial results, Panel B presents the merging analyses of the adjacent clubs, while Panel C shows the final results. The “PS” column values are the results of the estimation of Equation (3) for the different combinations of provinces, with the values in parentheses reflecting the log-ratios and the values above them to estimate the parameter *β* in (3).

**Table 3:** Factors driving the clubs.

		Marginal Effects			
Variable	Estimations	Club 1	Club 2	Club 3	Club 4
Travelers	−2.54 × 10^−5^ (−5.27)	8.94 × 10^−7^	5.10 × 10^−6^	−5.49 × 10^−6^	−5.08 × 10^−7^
Employed people in agricultural sector	−0.12 (−6.06)	0.004	0.024	−0.026	−0.002
Employed people in industrial sector	−0.288 (−11.96)	0.010	0.058	−0.062	−0.006
Central and South American immigrants	1.84 (4.87)	−0.065	−0.370	0.397	0.037
Life expectancy at birth	1.48 (4.10)	−0.052	−0.298	0.321	0.030
Capital of the region	−0.848 (−2.55)	0.030	0.170	−0.183	−0.017
Cut-points					
Cut-point 1	117.45				
Cut-point 2	121.20				
Cut-point 3	124.59				
N	51				
Pseudo R^2^	0.34				
Correctly classified cases	69%				
Brant statistic	7.09				

This table shows the coefficient estimates of the ordered logit model, with the *t*-ratios appearing in parenthesis. These were obtained by using cluster robust standard errors. The Brant statistic tests the null hypothesis of odds ratios proportionality and asymptotically follows a χ^2^ of (J-2)p degrees of freedom, with p and J being the number of explanatory variables included in the estimated model and the number of outcomes considered in the dependent variable, respectively. Columns 3–6 reflect reported marginal effects calculated at mean values for the estimated model.

## Data Availability

We used the Escovid19data database for daily confirmed case counts of the Spanish 50 provinces and 2 self-governing cities for the period in our study. This is available at https://github.com/montera34/escovid19data (accessed on 9 December 2020) [34].

## Appendix A

**Table A1:** Explanatory variables considered.

Definition	Unit	Source	Club 1	Club 2	Club 3	Club 4
Number of travelers to the province (average value of the period July–November 2020)	Travelers	INE [94]	58,902	71,343	52,818	77,932
Population density, 2020	Inhabitants per km^2^	INE [94]	721	111	135	115
Population in cities lower than 50,000, 2020	%	INE [94]	4.0	14.6	15.2	12.6
Employed people in the agricultural sector, 2020, Q4	%	INE [94]	9.4	6.9	6.0	4.9
Employed people in the industrial sector, 2020, Q4	%	INE [94]	11.3	17.2	15.6	11.8
Employed people in the construction sector, 2020, Q4	%	INE [94]	5.5	7.1	7.2	7.5
Employed people in the service sector, 2020, Q4	%	INE [94]	73.8	68.8	71.3	75.8
Per capita GDP, 2018	€	INE [94]	19,843	24,416	24,989	22,458
Human Development Index (HDI), 2014	-	[95]	0.8	0.8	0.8	0.8
Maghreb population over total, 2020	%	INE [94]	1.8	2.5	1.7	1.9
African population over total, 2020	%	INE [94]	2.1	3.1	2.2	2.4
Central and South American population over total, 2020	%	INE [94]	1.0	2.3	2.6	3.2
Population greater than 65, 2019	%	INE [94]	18.4	21.4	22.8	18.1
Population between 16 and 30, 2019	%	INE [94]	16.3	14.7	14.3	16.1
Average life of the population, 2019	Years of age	INE [94]	42.8	44.8	45.7	43.1
Life expectancy at birth (LEB), estimation of the average age that population born in 2019 will be when they die	Years of age	INE [94]	82.1	83.4	83.8	83.7
Infant mortality rate (IMR), probability of deaths of resident children under one year of age per 1000 live births, 2019	10^−3^	INE [94]	3.6	2.5	2.5	3.5
Number of physicians per 100,000, 2019	Physicians per 100,000 inhabitants	INE [94]	499	510	584	504
Number of nurses per 100,000, 2019	Nurses per 100,000 inhabitants	INE [94]	33	25	41	54
IgG seroprevalence at the second half of November 2020 (2nd COVID-19 wave)	%	ENE-COVID [74]	–5.5	6.2	8.3	7.4
IgG global seroprevalence until November 2020	%	ENE-COVID [74]	7.3	8.7	11.1	11.4
Positivity test rate (PTR), 24–30 November 2020	%	[89]	10.8	10.9	8.1	7.5

INE, Spanish National Statistics Institute (*Instituto Nacional de Estadística* in Spanish); ENE-COVID, national seroepidemiological study of SARS-CoV-2 infection in Spain.
